# Psychiatry trainees' experiences of cognitive–behavioural therapy training in a UK deanery: a qualitative analysis

**DOI:** 10.1192/pb.bp.115.051565

**Published:** 2017-04

**Authors:** Amy Alice Carson, Sarah Emily Clark

**Affiliations:** 1 University of Leeds, Leeds, UK

## Abstract

**Aims and method** To explore core psychiatry trainees' experiences of cognitive–behavioural therapy (CBT) training by using interpretative phenomenological analysis of semi-structured interviews conducted with seven core trainee psychiatrists in Yorkshire and the Humber Deanery.

**Results** Four key themes emerged: (1) barriers to training; (2) guidance, with emphasis on the importance of supervision groups; (3) acquisition of new skills and confidence; (4) personal influence on the training experience.

**Clinical implications** Many trainees in Yorkshire have a positive experience of CBT training; however, some also experience barriers to acquiring the relevant skills. Further research should build on the positive factors and barriers identified here, with a view to guiding improvements in training nationwide.

In 2009 the Royal College of Psychiatrists revised the curriculum for psychiatric training to include specific psychotherapy elements within the core curriculum for all psychiatry trainees.^1,2^ The curriculum outlines general and specific competencies.^2^ To achieve these competencies, trainees must complete a psychotherapy Assessment of Clinical Experience (ACE) as a workplace-based assessment (WPBA), attend case-based discussion (CBD) groups and undertake two psychotherapy cases in two modalities.^2^ This revision was an attempt to ensure that core trainees get sufficient experience of psychological treatments to be able to utilise them accurately, evaluate their effects intelligently and, where appropriate, deliver them competently. Cognitive–behavioural therapy (CBT) is one of the recommended modalities of psychological treatments. Psychiatry trainees are expected to be competent to deliver CBT effectively, and CBT is a valuable skill that enriches and informs their practice.^3–5^

The current literature suggests that prior recommendations for training in psychotherapies have not been widely implemented,^6–8^ despite trainees expressing an interest in acquiring such skills.^8^ Historically, trainees have been unaware of new curriculum guidelines^7–9^ and barriers to the training have been identified. These include lack of supervision,^10^ limited availability of suitable cases for training,^9^ and lack of protected learning time.^4,11–16^ A small study^17^ suggested working with low-complexity patients in the primary care setting as a way of overcoming these barriers, with protected time to do so. However, this has not been trialled on a wider scale, and limited data are available about whether this is a preferred route of training.^17^ Indeed, little qualitative research exists on the experiential accounts of psychiatric trainees undergoing the current training programme.

## Aims

This study aims to explore the experiences of CBT training, delivery and supervision in core trainee psychiatrists who have trained in Yorkshire and the Humber Deanery. This work aims to identify barriers that lead to negative experiences of CBT training and identify the factors that facilitate a positive training experience. We also hope this study will guide improvements to CBT training for core trainee psychiatrists to enable trainees to meet the Royal College of Psychiatrists' objectives and, ultimately, become more competent and psychologically minded psychiatrists.

## Method

This was an exploratory, qualitative study. Semi-structured telephone interviews and face-to-face interviews were used for data collection, and interpretative phenomenological analysis (IPA) was employed. IPA is a qualitative tool that enables the exploration of an individual's perception of events and how they ‘make sense’ of experiences.^18,19^ Approval for the study was granted by the Research and Development Department, Faculty of Medicine and Health, University of Leeds.

### Recruitment

Twelve core trainee psychiatrists in Yorkshire who had recently completed CBT cases under supervision were invited to participate in the study via email. One trainee declined participation as they were no longer working in the area and four trainees did not respond to the recruitment emails. Seven participants consented to take part in the study.

### Participants

The participants were a small, purposeful sample of seven core trainee psychiatrists (CT2 and CT3). Three participants were male. Two participants had worked in a psychotherapy post and one expressed an interest in specialising in psychotherapy.

### Procedure

The semi-structured interviews were guided by a review of the pre-existing literature concerning psychotherapy training experiences. This was piloted and amended as necessary. The questions concerned core trainees' experiences of CBT training and the generalisability of acquired competencies, the feasibility of training, the impact of supervision, the emotional aspects of training, and understanding of the College's curriculum. The interviews lasted between 12 and 24 min and were recorded. The recordings were transcribed verbatim to produce the raw data for the study.

### Analysis

The process of IPA^19^ was conducted by both interviewers, who listened to interview recordings and read and re-read the transcripts to familiarise themselves with the data. Initial interpretations of the data (descriptive summaries and points of interest) were entered as annotations in the margins of the transcripts. Emergent themes were then drawn and recorded in a table after cross-checking them against the transcript. This process was repeated for each transcript. Initially, the researchers searched for connections between the themes within the transcripts, and then between the transcripts. Thematic connections were identified and emergent themes were clustered, and a list of subordinate themes was compiled. Subordinate themes were clustered into superordinate themes. The superordinate themes and sub-themes were verified against the transcripts to ensure that they were grounded in evidence and then they were organised into a master table (Table 1). Last, quotations which best encapsulated each theme were identified.

**Table 1:** Themes

Superordinate themes	Subordinate themes
1. Barriers	a. Time b. Cases c. Patients d. Access to learning resources

2. Guidance	a. The role of supervision b. Learning from peers c. Sticking to the curriculum

3. Acquisition	a. Gaining new skills b. Models of mental illness c. Personal influence d. Confidence

## Results

Three superordinate themes were identified, with three or four subordinate themes each, and are described below.

### Barriers

Barriers to CBT training and the impact that this had on learning was a prominent theme across all of the interviews. Some participants had personally experienced barriers to training and others described the impact that barriers had had on their peers.

#### Time

The impact of insufficient time for learning was a theme that occurred across all seven interviews. Participants disclosed how shift work caused disruption to their CBT training.

‘if we were on-call that day or on nights or on leave, um, you ended up missing some of the sessions because of that and I think because CBT is quite structured, so you learn one thing in one session and then progress on to the next bit in the next session, so if you miss a bit I think it becomes a bit more difficult to get your head around it’ (participant 2).

One participant described the challenges encountered in maintaining protected learning time amidst other work commitments:

‘Because I was working in the ward […] it's quite difficult to get out of that to get supervision and do other things’ (participant 6).

Conversely, another participant perceived that colleagues respected protected learning time, suggesting that trainees' experiences varied depending on where they were working:

‘There's no problem getting time away from your day job to go [to] the CBT training’ (participant 7).

One participant, who had worked in a psychotherapy job, acknowledged that this job provided more time for training than other jobs:

‘I did a psychotherapy job in my last 6 months, so I was able to spend a lot more time and pick up a lot more cases than perhaps other people have experienced’ (participant 4).

One participant expressed concern that lack of time may prevent the use of CBT in future practice:

‘depending on the workload you're not always able to provide the adequate time per patient’ (participant 2).

The time delay between beginning CBT training and being allocated a patient case was identified as a barrier.

#### Cases

Participants felt that there was a shortage of CBT cases available for trainees and this had a significant impact on their CBT training.

‘I think the barrier is there is a big waiting list and that is a problem, like you have to wait to get a case […] and obviously if there [aren't] enough patients […] you [won't] get a case and there's loads of trainees and this is kind of a mandatory thing, everybody has to have a case’ (participant 3).

Participants felt that it would be beneficial to have more than one case:

‘I would say if we had at least two cases that might be better but again it is very difficult to get a CBT patient because we have got loads of trainees and, um, not a lot of CBT we do, we get for trainees because […] if it is not simple for trainees they do not consider it, to give it to us, and there is a long waiting list, I think you know that, there is a big waiting list for CBT, so like I had to wait for nearly … more than a year actually’ (participant 3).

A participant who had worked in a psychotherapy post observed that one case would not have provided sufficient CBT experience:

‘I work in psychotherapies so I have had more than one case and [I] have had good exposure to CBT. I just don't know whether if I hadn't had this job, this exposure would be enough’ (participant 1).

#### Patients

Six of the participants discussed the impact of the patient on their experience of CBT. First, participants felt that completing a CBT case was more challenging if the patient did not engage. Second, they discussed the impact of patients not attending sessions or discontinuing with treatment; this was perceived to be a substantial barrier to training that was not taken into account by the College's curriculum.

‘if the patient doesn't engage or doesn't complete therapy – this could potentially be a problem. In fact [the trainee] might not have the time to undertake another case and complete it’ (participant 7).

However, participants acknowledged that the impact of the patient on CBT training was a factor that is difficult to control.

‘It's totally up to the [patient] whether he or she will continue or not and if she leaves before you complete the full therapy then you have to wait for the next patient so that is a problem but I don't know the way to change it because it's totally up to the patient if they will continue or not’ (participant 3).

Conversely, one participant acknowledged that a good doctor-patient relationship could have a positive impact on the experience of CBT training.

‘seeing results from patients as well has been really good’ (participant 4).

#### Access to learning resources

Participants expressed concern about keeping skills up to date as time elapsed.

‘I think the Royal College [of Psychiatrists] run a CBT module, but it's all things that you have to pay for […] and I think that most people feel like they pay for enough exam material [and] for the Royal College exam, and probably don't have [a] mountain of spare cash to be spending on more e-learning stuff, so it might be good if the trust wanted to sort of do something with CBT, or if the Royal College will give out [an] e-learning module – I think that would be quite useful, and for people who are not seeing cases that regularly – I think it might kind of just update you with CBT’ (participant 4).

Conversely, others thought that the time and experience was ‘ample’:

‘I've been given adequate texts to read and stuff in my spare time. I have ample opportunity to discuss any complications that arise with my case so […] all in all it's been really good’ (participant 2).

### Guidance

The participants perceived that supervision was an important feature within their experience of CBT training. The superordinate theme of guidance was identified across all interviews, with the subordinate themes of supervision, peer learning and curriculum.

#### The role of supervision

There was an overall satisfaction with supervision from all the participants, who felt that they had continued support and advice. Feedback and reassurance from supervision encouraged the trainee to gain confidence and it was highlighted that the participants felt able to ask their supervisor for advice.

‘I would say that the supervision was really good, it was tailored down to trainees' need […] the supervisor was approachable’ (participant 5).

‘there were quite a few things that needed improvement and I felt that supervision enabled me to identify these areas and work on improving these sets of things’ (participant 1).

It appeared that the expertise of the supervisor themselves was respected and was useful to the majority of the participants. All of the participants had a consultant psychiatrist as their supervisor.

‘expert opinion on where you are going with your cases, so you feel like you do a good job with the patient’ (participant 4).

‘has a lot of experience on this ground so that was quite helpful’ (participant 3).

In terms of emotional support, there appeared to be a consensus that, if required, emotional support from supervision would be present.

Interviewer: ‘And, do you feel like you had enough emotional support if needed during your training?’

Participant: ‘I suppose I would, yes. It was never an issue, but I would imagine that if I had felt stressed I would have found support’ (participant 1).

#### Learning from peers

Three of the participants discussed how helpful peer learning was in their training, in particular as regards case supervision conducted in group sessions.

‘Well, I actually used to love and look forward to […] supervision, because every time – because our supervision was a group sort of supervision – I learn not only from my case, but [I] also learn from other people's cases. Because people have different aspects they need supervision [for], so I will kind of learn quite generally because it's quite enjoyable to keep on listening to different cases, including mine – and following it up through week after week. So I really enjoyed it’ (participant 6).

#### Sticking to the curriculum

In contrast to the optimism surrounding supervision, the participants did not consider the College curriculum to be a sufficient source of guidance.

I: ‘Also, how aware were you of the Royal College guidelines before you started your CBT training?’

P: ‘Um … not very.’

I: ‘And do you feel that there is any way in which they could be accessed more easily?’

P: ‘I wouldn't even know how you access them now to be honest.’

I: ‘Okay, that's okay. Okay.’

P: ‘I'm assuming that you look on the Royal College website but I never have’ (participant 7).

This lack of awareness of the Royal College of Psychiatrists' curriculum guidelines was found in other participants, who reasoned that the guidelines are too extensive and incomprehensible. However, it transpired that six participants had acquired the competencies outlined in the curriculum despite the fact they were unaware of what these were.

‘The curriculum for core training is huge and extremely vague mostly – so you need to trawl through that document, probably most people haven't’ (participant 4).

P6: ‘I've been able to explain to the patient what CBT is and what it is used for and also, sometimes I've been able to use the skills I've learnt in CBT, to offer treatment to the patient.’

I: ‘Do you feel like you are able to deliver CBT?’

P6: ‘I think I feel that way’ (participant 6).

One participant relayed that the curriculum needed to be more flexible owing to the nature of the therapy itself being unpredictable and time consuming.

‘I do think they need to be a bit flexible, because say if a patient drops out of therapy and say you have done 10 sessions that now doesn't count as a case!’ (participant 4).

### Acquisition

The participants felt that they acquired a great deal via their CBT training, in terms of gaining specific CBT skills, but also in learning generic skills that could be applied to psychiatric practice and learning which patients would be suitable for CBT. They acquired a new insight into models of mental illness and learnt to conceptualise mental illness in accordance with the CBT model. Trainees also discussed their personal influence on their experience and thus their acquisition of CBT skills. Last, they grew in confidence as they gained experience working with their CBT case.

#### Gaining new skills

The trainees felt that they gained a great deal from their CBT training, in terms of both specific CBT skills and also broader transferable skills that could be applied to their psychiatric practice.

‘I enjoyed it. I think basically it's really important […] for [a] psychiatric trainee or for a psychiatrist to have experience in CBT’ (participant 1).

Most of the participants felt that they gained a greater understanding of what CBT entails and how it works. This enabled them to confidently explain CBT to patients.

‘it gave a clear understanding for me of what exactly CBT involves and how it has a beneficial effect on patients’ (participant 5).

Three participants described how the training helped them to identify which patients would be suitable for CBT. They felt confident in referring patients for CBT. However, others felt that they needed more experience to accurately assess patients for CBT.

‘I mean, now like when I will refer patients for psychotherapy I would now know what are the categories that I need to check before referring and whether the patient is suitable for CBT or not because I have practical experience of doing it and I know that I've some idea whether the patient [would benefit] from CBT or not’ (participant 3).

Some of the participants felt confident using CBT techniques. However, they acknowledged that they had limited experience and that they were not fully equipped to deliver formal CBT.

‘And do you feel like you would be able to deliver it as well?’ P: ‘Delivering, to be honest – no. Because, I think, err, having done only one short case of CBT, without any supervision, I won't be able to take up a case on my own I guess … ’ (participant 5).

Although not all of the participants felt confident in delivering CBT, they felt that they had gained transferable skills that could be used elsewhere in their psychiatric practice.

‘Sometimes in my session now […] I see people with […] anxiety and other disorders; I am able to use the very same skills I used in my CBT session to kind of handle the situation around me’ (participant 6).

The participants hoped that they would continue to use the skills that they had gained. However, some expressed concern about losing skills over time, particularly if they did not use CBT regularly in their job.

‘if you're not in touch then you may lose some skills. That may be a problem in the future because you're not going, not actually keeping doing it, practising it, yeah, so maybe it can impact on practice in the future’ (participant 3).

#### Models of mental illness

Six participants talked of the training causing a shift in their understanding of mental illness, moving them away from the diagnosis exclusively, and focusing on the wider problems for their patient, allowing them to reach the criteria of the curriculum and develop their emotional intelligence.

‘what I found out is that [pause] maybe some of these people do not have defined mental illness but they definitely have a problem, and just basically move me away from having to diagnose a patient with something, so [I was able to] focus on the problem rather than the diagnosis, and sometimes the problem did not correspond to an ICD-10 diagnosis, and I think this is really useful because, eh, usually in everyday life, people have problems – rather than psychiatric diagnosis’ (participant 1).

‘Well it has given me the insight into looking at the behaviour and thoughts, in terms of how people are affected, and how to help them – that's not what I was thinking before, because before I was thinking in terms of the medical model, and now I'm thinking more about other things like their thoughts and their behaviour, and their emotion – and how all of that is part of their illness, and how to use that to treat their illness’ (participant 6).

The trainees described how this increased awareness affected the management of their patients.

‘it does change your thinking about your practice, and you know – what else is out there, other than, you know, medication and that kind of thing, there are other ways that people can benefit from secondary care’ (participant 4).

‘having done CBT training [pause] it helped me to identify that there are some mental disorders which need both medications and psychotherapy’ (participant 5).

The benefits of having time to reflect as part of psychotherapy training gave trainees a different perspective on the patient that they were treating.

‘and I think when you're using CBT to make them think differently about their illness and their actions it makes you think differently about it as well […] and you certainly see patients' difficulties from a different point of view […] and it gives you time to figure that out’ (participant 2).

Personal use of this new way of looking at models of mental illness was cited; the participants described how this changed how they see themselves.

‘within myself, it changed me in such a way, the way I am able to kind of evaluate my behaviour, with what I do and what I think – so I use it on myself quite a bit. If I find myself in a difficult situation, even in day-to-day life – I use the same principle on myself to kind of look at how things are done, and change things differently. So I think that's how personally CBT has influenced me’ (participant 6).

#### Personal influence

Several of the participants acknowledged that they had a particular interest in CBT. Two had worked in a psychotherapy post and one hoped to specialise in psychotherapy. Furthermore, the participants acknowledged that their personal interest may have affected their experience of CBT training and they may have gained more from the training as a result.

‘Personally, I am interested in psychotherapy anyway, so I wanted – I want to be able to use CBT [pause] later on in my career, so [pause] so that's one of the reasons why I think it was really useful’ (participant 1).

#### Confidence

The majority of the participants talked of increased confidence during their training and afterwards. This is in regards to their own skills and understanding, as well as recognition of when to refer a patient for CBT.

‘since my first case, [I] have got a lot more fluent [than] in the beginnings of therapy’ (participant 4) .

One participant expressed a lack of confidence in referring patients for CBT because their CBT supervision was still ongoing at the time of the study:

‘I don't think I'm confident at the minute because I suppose I've been given a patient, I've not assessed someone for it as such but, um, I'm continuing to have CBT supervision […] so I think by the end of it I will be able to, yes, to figure out who would benefit from it’ (participant 2).

Overall, there was a positive association between experience and confidence.

‘Do you know, I feel much more confident about CBT … because I know what it is, so I feel much more confident’ (participant 3).

## Discussion

A number of barriers that affect trainees' experiences of CBT training have been identified here; chiefly a lack of protected learning time, a shortage of available cases for training purposes and difficulties arising due to problems with patient engagement and therapy completion.

Having protected time for CBT training was high-lighted as crucial for psychiatry trainees, who reasoned that the difficulty in completion and the formulaic structure of CBT require a regularity and dedication to carry it through. The ‘inevitability’ of work disruptions and shift patterns were the main source for these disruptions recognised in this study. As previous work suggests, there was variability within this, dependent on where one is a trainee and what jobs one is assigned. Trainees in a dedicated psychotherapy post were more positive about their ability to complete and transfer their CBT skills. We propose that this may be due to the trainee having a personal interest in ‘talking therapies’ in addition to the granted protected time to acquire these skills in a psychotherapy post, a proposition which resonates with previous work in this area.^4^ This variation resulting from chance permeates to the level of patient allocation as well – as each trainee is allocated a different patient, standardisation of experience becomes problematic. One aspect that helped with this was peer-group learning, insofar as the experience of each trainee is shared and hence multiple cases are acquired instead of just the one that each trainee has had. This echoes previous recommendations to utilise novel ways, such as peer-group learning, to assist CBT supervision and skills acquisition.^3^ Thus, ensuring that protected learning time is provided and that it is a feasible task is likely to improve the trainees' experience.

Concerning the shortage of cases, further enquiry would be beneficial to explore the feasibility of targeting the long waiting lists for both the patients and the trainees by enabling trainees to take on a broader range of patient cases. A larger study would be beneficial in exploring this, continuing with the idea to source cases from primary care.^17^ This could help to relieve the pressure for the trainee to complete one ‘ideal’ CBT case, and thus the patient being a barrier to learning could have less impact. It was also suggested that it could be beneficial to provide a follow-up course that can be accessed freely to ensure that skills are maintained over time. Furthermore, the training experience could be improved if the Royal College of Psychiatrists' curriculum took into account the effect of patients discontinuing with therapy and allowed a degree of flexibility for cases in which almost all sessions had been completed. Moreover, as prior literature suggests, further dissemination and accessibility of the College curriculum is still warranted.

A number of factors that facilitated a positive experience of CBT training in Yorkshire were identified. Supervision was highly valued and deemed to be an important facilitating factor during the training. Further research could be useful in order to elucidate how the benefits of supervision are mediated and thus enable similar supervision to be conducted elsewhere.

In accordance with the College curriculum, this study suggests that psychiatric trainees in Yorkshire report enhancements in their emotional intelligence and being able to refer for CBT accurately and evaluate its effect intelligently after the training. A broad positive association was relayed from the participants between exposure to CBT and confidence in recognition and delivery of skills learned. However, although trainees' overall confidence about psychotherapy increased, further experience is deemed necessary for the trainees to feel able to deliver CBT competently. On the whole, trainees gained a broader perspective of models of mental illness and learned transferable skills, which have now influenced their clinical practice.

Although a robust study design was employed and triangulation of the data was used to increase the validity of the findings, the qualitative nature of the study has inherent limitations.^20^ Nonetheless, the study provides a valuable insight into the experiences of psychiatry trainees in Yorkshire and paves the way for further research in other deaneries across the UK in order to gain a clearer insight into the experiences of core trainee psychiatrists in general, with the aim of improving CBT training and ultimately enabling psychiatrists to become more emotionally aware, competent and confident.
